# Community Pharmacists’ Motivation and Barriers to Providing and Billing Patient Care Services

**DOI:** 10.3390/pharmacy8030145

**Published:** 2020-08-14

**Authors:** Liesl D. Reyes, Jenny Hong, Christine Lin, Jeffrey Hamper, Lisa Kroon

**Affiliations:** 1 Albertsons Companies, Pleasanton, CA 94588, USA; reyes.ldm@gmail.com (L.D.R.); jenny.hong@safeway.com (J.H.); christine.lin@albertsons.com (C.L.); jeffrey.hamper@albertsons.com (J.H.); 2 School of Pharmacy, University of California, San Francisco, CA 94143, USA

**Keywords:** community pharmacist, scope of practice, patient, care, services, billing, AB 1114

## Abstract

Recently, California (CA) pharmacists’ scope of practice has expanded to include independently prescribing self-administered hormonal contraceptives, nicotine replacement therapy medications, travel health medications, routine vaccinations, naloxone hydrochloride, and HIV preexposure and postexposure prophylaxis. However, previous reports indicate that practicing within this expanded scope has remained limited. Therefore, a 26-item, web-based survey was emailed to CA community pharmacists to assess pharmacists’ knowledge, intent, and barriers to prescribing and billing for these patient care services. A total of 216 chain, supermarket-based, independent, mass merchant, and health-system outpatient pharmacists were included. The primary services provided and medications prescribed are for vaccinations and naloxone. Most pharmacists agree that engagement in and implementation of new strategies to enhance patients’ access to care is important. Common barriers include patient unawareness of pharmacist-provided services, lack of payment for services, and difficulty incorporating services within pharmacy workflow. Pharmacists are confident in their ability to provide patient care services but are less knowledgeable and confident about billing for them. Enhancing promotion of pharmacist-provided services to patients, developing strategies to efficiently incorporate them into the workflow, and payment models can help overcome barriers to providing these services.

## 1. Introduction

Community pharmacists across the United States have had their scope of practice expanding for the delivery of patient care services beyond the clinical care involved through medication dispensing, including authorities to prescribe medications and order tests. In California (CA), several recent bills were passed that expanded all CA licensed pharmacists’ scope of practice to perform the following [1,2,3]:

1. Independently furnish self-administered hormonal contraceptives, nicotine replacement therapy medications, and medications for symptoms not requiring a diagnosis that are recommended for international travelers (Senate Bill (SB) 493)
2. Furnish naloxone hydrochloride in accordance with standardized procedures or protocols (SB 1535)
3. Furnish HIV preexposure and postexposure (PrEP/PEP) medications (SB 159)

Note that although SB 159 was approved, the regulations for the required training were pending at the time of this study.

Pharmacists may furnish the medications mentioned above, contingent upon completing approved education training. In California, the term *furnish* is defined as “to supply by any means, by sale or otherwise, and essentially means prescribe; the term prescribe will be used in this paper [4]. SB 493 also provides eligibility for the appointment of an Advanced Practice Pharmacist (APh). APhs can perform patient assessments, order and interpret drug therapy tests, refer patients to other health care providers, perform disease state management, and practice under collaborative practice agreements. Pharmacists that complete at least two of three of the following may be licensed as an APh [5]:

1. Earn certification in a relevant area of practice, including, but not limited to, ambulatory care, critical care, geriatric pharmacy, nuclear pharmacy, nutrition support pharmacy, oncology pharmacy, pediatric pharmacy, pharmacotherapy, or psychiatric pharmacy, from an organization recognized by Accreditation Council for Pharmacy Education (ACPE) or another entity recognized by the board.
2. Complete a postgraduate residency through an accredited postgraduate institution where at least 50 percent of the experience includes the provision of direct patient care services with interdisciplinary teams.
3. Have provided clinical services to patients for at least one year under a collaborative practice agreement or protocol with a physician, advanced practice pharmacist, pharmacist practicing collaborative drug therapy management, or health system.

Assembly Bill (AB) 1114 took effect on 1 April 2019 and provides pharmacist payment for patient care services provided to California Medicaid (Medi-Cal) beneficiaries [6]. Pharmacists are eligible for up to 85% reimbursement of the fee schedule for physician services. This is contingent upon the pharmacist enrolling as an ordering, referring, and prescribing (ORP) provider. 

SB 493 also included the designation of pharmacists as health care providers, which is often a necessary step for insurers to pay for health care services. Unfortunately, on the national level, United States pharmacists do not have provider status with the federal government. Despite these progressive steps for CA pharmacy practice, APh licensure and reported furnishing rates have remained limited. As of 15 January 2020, there were 624 APhs in CA of approximately 47,670 licensed pharmacists (1.3%). A study assessing CA pharmacy practice revealed that after two years (2016) of implementing the statewide protocol for pharmacists to prescribe naloxone, only 23.5% of chain and independent community pharmacies offered services to prescribe naloxone [7]. Furthermore, only 50.6% of the pharmacies that offered naloxone prescribing had naloxone in stock. In addition, the February 2019 California Health Report reported that 15% of pharmacies offered prescribing of self-administered hormonal contraceptives, with few women having knowledge about pharmacists’ ability to provide this service [8]. While the expanded scope of practice laws is a significant achievement, implementation is even more critical for pharmacists to deliver and improve patient access to these health care services.

Though previous research has quantified the level of specific medications being prescribed by pharmacists, there is limited data about pharmacists’ perceptions of their experiences in implementing services within the state of California. This study assesses pharmacists’ motivations, perceived barriers, and level of engagement in expanded patient care services and associated prescribing of medications.

## 2. Materials and Methods

### 2.1. Study Design

A web-based survey was distributed to community pharmacists throughout California. Pharmacists that practice within chain, supermarket-based, independent, mass merchant, and health-system outpatient pharmacies were eligible to complete the survey. The study was approved as an exempt study by the University of California San Francisco Institutional Review Board (IRB). Per the IRB requirements for survey-based research, each question was optional. Therefore, the total number of respondents could differ for each item. Participants were ineligible if they identified themselves as a health-system inpatient or ambulatory care pharmacist. These individuals were directed to the end of survey. Participants who completed at least 75% of the survey were considered eligible for study analysis, and thus, able to participate in an optional incentive. The incentive consisted of a raffle for an Albertsons Companies gift card. Participants provided their preferred method of contact (e.g., email and/or phone number). One $100, one $75, one $50, and three $25 gift card winners were randomly selected and emailed to accept their prize.

### 2.2. Web-Based Survey Development

Survey questions were categorized by knowledge, motivation, and barriers to providing patient care services. Demographic information such as pharmacy training and experience, pharmacy setting, and pharmacy patient population was collected. The 26-item survey was developed using Qualtrics^®^ software (Provo, UT, USA) and comprised Likert scale, multiple choice, and free text response questions. Likert scale items that contained multiple questions were considered one question, which consisted of five levels of agreement: strongly disagree, disagree, neutral/neither agree nor disagree, agree, and strongly agree. Items regarding self-reported provision of patient care services and prescribing of self-administered hormonal contraceptives, nicotine replacement therapy medications, travel health medications, routine vaccinations, and naloxonewere included. To assess for knowledge of which medications can be prescribed and to assess if a participant carefully answered each question, three distractor options were included within the question that asked this. Distractor options in multiple choice questions are incorrect answers that would be selected by individuals with partial knowledge but ignored by those with full knowledge of a subject [9]. Bupropion, varenicline, and HIV PrEP/PEP medications were included as distractor options. Bupropion and varenicline are not considered nicotine replacement therapy medications, and HIV PrEP/PEP medication prescribing has not been implemented yet. Questions were peer-evaluated by two University of California, San Francisco faculty members for content validity and refined prior to distribution to pharmacists.

### 2.3. Electronic Survey Distribution

The web-based survey was emailed to California community pharmacists through the California Pharmacists Association (CPhA), the California Community Pharmacy Enhanced Services Network (CPESN), and Albertsons Companies pharmacies. CPhA is the primary state professional association representing community pharmacists, with approximately 3800 members. CPhA members practice within various community-based pharmacies, and pharmacists in the CA CPESN practice primarily in independent pharmacies. Albertsons Companies distributed the survey to all pharmacists within their Northern and Southern California divisions (Albertsons, Safeway, and Vons locations). Survey distribution occurred between 28 February 2020 and 31 March 2020 on five dates: February 28 (CPhA), March 1 (Albertsons Companies, Northern California Division), March 2 (Albertsons Companies, Southern California Division), March 13 (CPESN, CPhA), and March 31 (CPhA). Data collection continued until 17 April, 2020.

### 2.4. Data Analysis

Data was analyzed using descriptive statistics and cross-tabulation through Qualtrics^®^ software and Microsoft Excel^®^ (Redmond, WA, USA). Though 216 participants’ surveys were included for analysis, the total number of respondents (i.e., denominator for items) varied per question since each was optional. Pharmacists’ motivation, barriers, and level of engagement in providing patient care services and pharmacist and pharmacy demographics were evaluated.

## 3. Results

### 3.1. Participant Characteristics

**Figure 1:**
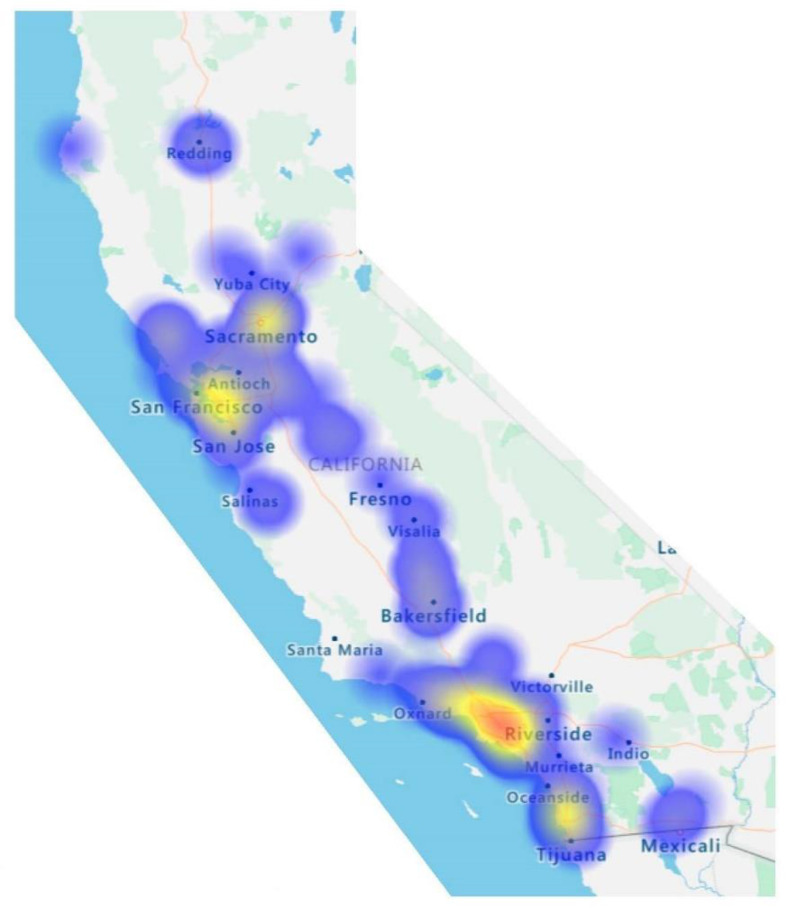
Geographic distribution of survey participants.

A total of 255 pharmacists started to take the survey. Of the 255 respondents, 216 were community-based pharmacists that completed at least 75% of the survey and were therefore eligible for study analysis. Figure 1 highlights respondents’ geographic locations, and Table 1 depicts respondents’ demographics. Of the respondents, 78% (110/142) earned a PharmD degree and 22% (32/142) earned a B.S. Pharmacy degree. Some individuals completed postgraduate training, earned certifications, or became licensed as APhs (7%, 9/32). While respondents’ community pharmacy positions varied, over half of the 193 respondents were pharmacy managers and 54 were staff pharmacists. On average, respondents had been practicing pharmacy for 19 years (SD 15.4, range 1–59) and worked an average of 37 h per week (SD 13.8, range 5–70). Respondents were represented across California (Figure 1).

The pharmacy setting characteristics are shown in Table 2 and Table 3. A majority of pharmacists practiced within independent pharmacies (41%, 87/210) or grocery store pharmacies (33%, 70/210). Fewer pharmacists worked in chain (16%, 32/210), health-system (4%, 8/210), or mass merchant (1%, 2/210) pharmacies. Participants were asked to approximate the percentage of individuals that corresponded to the patient population they served. Pharmacists indicated serving populations primarily consisting of middle-age adults (40–64 years old) or older (65 years and older), at 39% (72/183) and 59% (106/179), respectively. The appointment-based model was used by 25% (38/152) of pharmacists.

### 3.2. Knowledge of Patient Care Services

Table 4 shows three multiple choice questions that assessed pharmacists’ knowledge of provision and billing for patient care services regarding self-administered hormonal contraceptives, nicotine replacement therapy medications, travel health medications, routine vaccinations, and naloxone. Less than one-fourth (34/168) of participants answered all questions correctly. Most of these individuals were pharmacy managers, earned a PharmD degree, or worked in independent community pharmacies.

### 3.3. Motivation to Provide Patient Care Services

**Figure 2:**
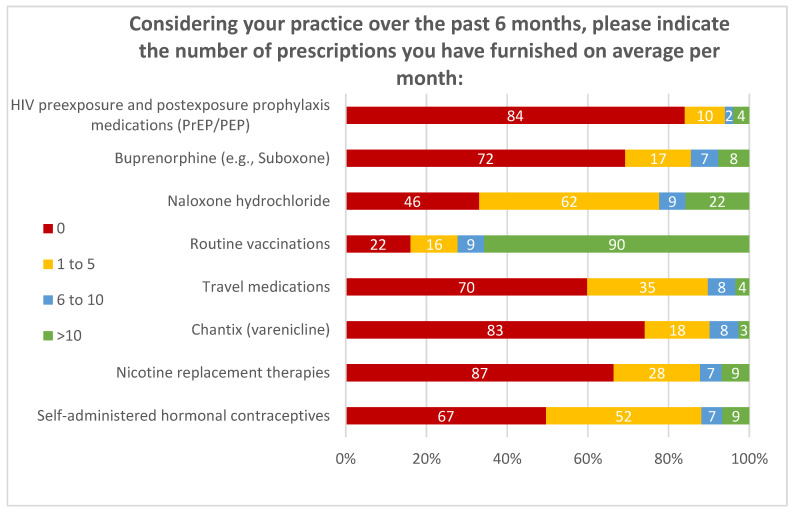
Number of medications prescribed in the previous 6 months.

**Figure 3:**
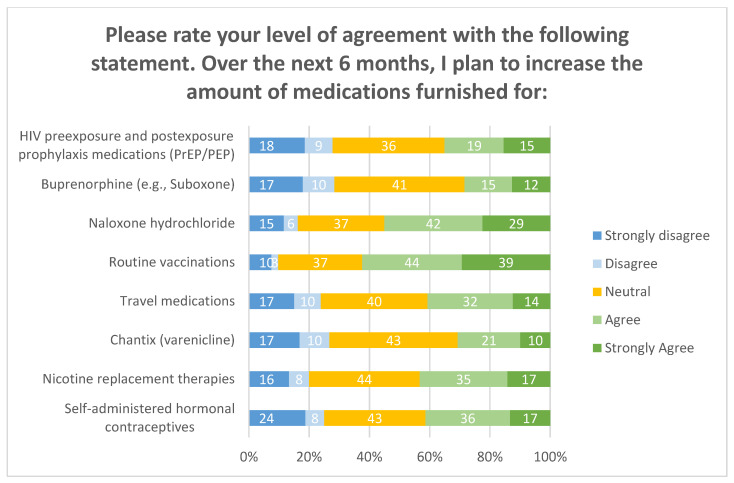
Intent to increase medication prescribing.

Respondents were asked if they have completed the necessary training for furnishing self-administered hormonal contraceptives, nicotine replacement therapy medications, travel health medications, routine vaccinations, and naloxone. As previously mentioned, bupropion, varenicline, and HIV PrEP/PEP trainings were included as distractor options. Though HIV PrEP/PEP furnishing authority has been approved, trainings had not been implemented at the time of the study and therefore pharmacists could not prescribe. A majority of pharmacists indicated that they had completed the trainings for routine vaccinations and naloxone, at 72% (122/169) and 65% (110/169), respectively. Although bupropion and varenicline were included as distractor options, and with HIV PrEP/PEP trainings not implemented yet, nine individuals reported that they have completed them. Figure 2 and Figure 3 depict the average number of medications prescribed in the previous 6 months and a pharmacist’s intent to prescribe in the next 6 months. Since a majority of pharmacists have completed trainings for routine vaccinations and naloxone, most were prescribing >10 routine vaccinations (66%, 90/137) and naloxone (16%, 22/139) prescriptions monthly. However, for self-administered hormonal contraceptives, 67% (6/9) of APhs were prescribing 1–10 prescriptions per month.

**Figure 4:**
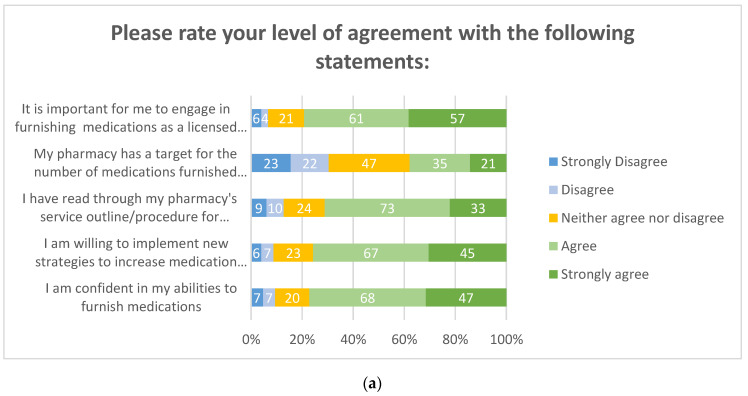

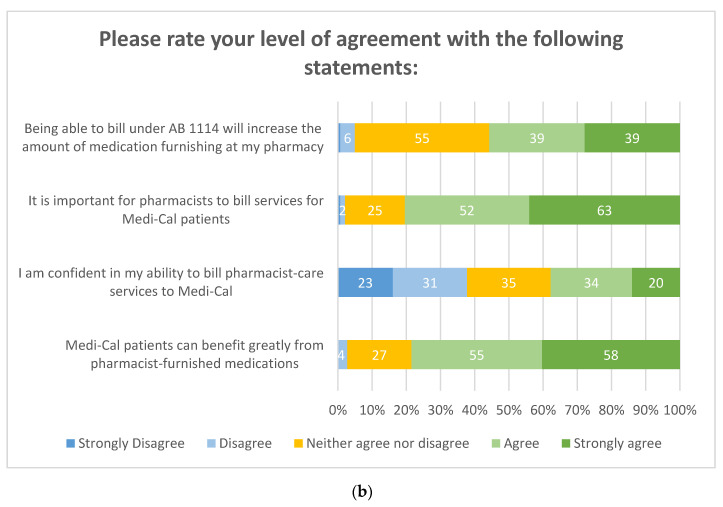
(**a**) Motivation to implement patient care services. (**b**) Motivation to bill for patient care services.

**Figure 5:**
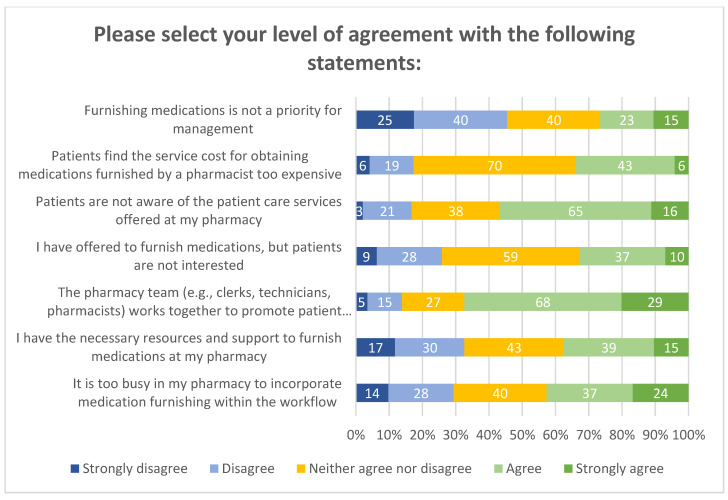
Barrier questions.

Overall, pharmacists agreed they intended to increase medication furnishing over the next 6 months, primarily for routine vaccinations and naloxone once again. Over half of respondents (77%, 115/149) were confident in their abilities to furnish medications, were willing to implement new strategies to increase medication prescribing, and have read through their pharmacy’s service outline/procedures. These items are depicted in Figure 4a,b and Figure 5. Approximately one-third of respondents reported that their pharmacies had a goal number of medications to prescribe per month (32%, 47/148).

Regarding services provided to state Medicaid beneficiaries, only 32% (48/149) were enrolled as ORP providers. The same number of individuals, 32% (48/149), did not know what an ORP provider was. Notably, a subgroup analysis revealed that 100% (38/38) of individuals enrolled as an ORP provider due to management requirements (38/48) worked in grocery store pharmacies. In addition, 100% (9/9) of those enrolled by their own initiative were independent community pharmacists. Twenty-three pharmacists indicated they did not intend to obtain their ORP provider status, for reasons that they are not interested at this time (36%, 8/22), management does not require it (32%, 7/22), or the process is too confusing (14%, 3/22). Overall, pharmacists agreed that Medicaid patients can benefit from pharmacist-prescribed medications, it is important to bill for their services, and the ability to receive reimbursement will increase the amount of medication prescribing. However, only 38% of pharmacists indicated they were confident in readiness to bill Medi-Cal for patient care service, and 38% indicated they were not confident, as depicted in Figure 4b.

### 3.4. Barriers to Providing Patient Care Services

Barriers to providing patient care services are shown in Figure 5. Fifty-seven percent of pharmacists reported patient unawareness of pharmacist-provided services. Forty-three percent of pharmacists indicated it is too busy in the pharmacy to incorporate services within workflow. The number of prescriptions dispensed per week among these respondents was between 500 and 1500. While some indicated that they work within busy environments, over half of respondents (97/144) indicated that their pharmacy teams work together to promote patient care services, and lastly, 34% of respondents perceive that patients find the cost for obtaining pharmacist-prescribed medications too expensive.

## 4. Discussion

### 4.1. Findings

A goal in expanding community pharmacists’ scope of practice is to increase patients’ access to care. With this, pharmacists must then implement these expanded roles. This study identified the factors that motivate CA pharmacists to do so and the barriers they experience within their daily practice.

Most pharmacists expressed that the provision of patient care services and associated prescribing of certain medications are important. The most prescribed medications are vaccinations and naloxone. Interestingly, APhs are prescribing a greater amount of self-administered hormonal contraceptives. Since licensure as an APh is self-initiated, it is likely that APhs feel more inclined to prescribe medications. Pharmacists (43%) indicated intent to increase medication prescribing over the next 6 months. We chose to include distractors for certain questions to assess if respondents were carefully reading the question. Unfortunately, 80% of respondents provided positive responses to these distractors, which possibly indicates they did not read the question carefully or lacked knowledge that pharmacists are not able to prescribe these medications. Of these, 9 individuals were licensed as APhs, 38 worked in grocery store chain pharmacies, and 66 in independent community pharmacies. Those that reported that they have completed trainings and plan to increase prescribing levels for the distractor answers bupropion and varenicline could have incorrectly thought these were grouped within nicotine replacement therapy. Additionally, the word “furnish” may have been interpreted as “dispense.” The word furnish was used in the survey to be consistent with the California legal terminology for pharmacists’ prescriptive authority.

Regarding ORP provider enrollment, all respondents who enrolled by their own initiative were independent community pharmacists, whereas those who enrolled due management requirements were pharmacists practicing in grocery store chain pharmacies.

Regarding services provided to Medicaid beneficiaries, 32% of pharmacists indicated lack of knowledge of an ORP provider. Since AB 1114 was implemented in April 2019, our findings show that many pharmacists are still unaware of how to implement billing Medi-Cal for patient care services. Lastly, the ability to bill for services under AB 1114 was a key factor reported for pharmacist engagement in the provision of these patient care services.

Our convenience sample size was 216, with smaller subgroups among each practice setting. Fewer than half of participants correctly indicated which medications pharmacists can prescribe at the time of the study. Pharmacy managers and independent community pharmacists were the primary respondents that answered correctly, possibly demonstrating their motivation to remain up-to-date with laws, as leaders of their teams, or to actively engage in providing such services. Additionally, not all pharmacists indicated that their pharmacy has a target number of medications to furnish per month. Having targets can provide structure for pharmacists to prioritize these services. However, it is notable that targets may also lead to additional pressure that will force pharmacists to provide these services rather than doing so from an internal motivation. If targets are implemented, it will be important for pharmacy teams to ensure involvement of all team members and consider appropriate staffing ratios.

Other studies have assessed pharmacists’ motivation and barriers to prescribing medications in other states. A systematic review conducted by Blalock et al. regarding delivery of patient care services revealed that reimbursement is “likely to increase pharmacist motivation to engage in service delivery” [10]. Pharmacists that were reimbursed for the services they provided yielded more effective delivery and positive patient interventions. Similarly, our findings revealed that with the ability to bill for services under AB 1114, over half of respondents agreed that they would increase medication prescribing. With pharmacists’ expanding scope of practice, it is important that they are appropriately compensated for services provided. Additionally, a study conducted in Canada, the first country to authorize pharmacists to prescribe medications, indicated that pharmacists in patient-focused pharmacies were more likely to adopt prescribing habits compared with those in product-focused pharmacies [11]. Interestingly, a majority of those respondents that are ORP providers in our study were independent community pharmacists. Given that independent pharmacies tend to have a lower prescription volumes compared with larger chain pharmacies and owners can make decisions autonomously without a corporate structure, it may be easier to engage in expanded patient care services. Though our study did not assess this, asking pharmacists if they perceive their environment to be “patient” versus “product” focused could have provided that insight. Also, strong physician relationships foster a likelihood for pharmacists to deliver patient care services. With this insight, it would have been beneficial to evaluate the level of participants’ physician relationships. It could be that those who were more likely to prescribe medications had more established relationships with physician prescribers. This highlights the importance of interprofessional collaboration in enhancing the provision of patient care.

For barrier to providing patient care services, Bakhireva et al. identified time constraints and the out-of-pocket patient fee for the service as important barriers to pharmacists prescribing naloxone in New Mexico [12]. Authors concluded that strategies to overcome these barriers are to focus on pharmacists’ concerns and improve patient education about benefits of the medication. Our findings are consistent with this study in that many pharmacists find that they are too busy to incorporate medication prescribing within their workflows. However, provision of services will also be contingent upon patient demand. Unless patients are aware of such services, uptake will continue to be low. Another study assessed pharmacist knowledge about naloxone and reported that several respondents incorrectly indicated that “naloxone was a controlled substance, that a tablet formulation was available, and that injectable formulations not appropriate for layperson use were available” [7]. The number of respondents was not disclosed. As mentioned previously, payment is a key motivating factor for a pharmacist to engage in medication-prescribing services. While CA is one of the states that has implemented legislation to authorize pharmacist reimbursement, other states have not due to pharmacists’ lack of recognition as providers. Newman and colleagues emphasized that because the Centers for Medicare and Medicaid Services (CMS) does not recognize pharmacist provider status, states’ “participation and uptake in these new payment models have been limited [13].” At the state level, some states have recognized pharmacist provider status and therefore have the opportunity to bill certain patient care services.

As with all health care services, pharmacists need to be educated before implementing such services. This can be done at the pharmacy level, and education will be specific to the institution in which pharmacists work. We found that many pharmacists have not completed the necessary trainings to prescribe medications other than vaccinations and naloxone. Therefore, the current level of providing the expanded patient care services and associated medication prescribing is low. Fortunately, many California pharmacy school curricula include content to educate students on pharmacists’ expanded scope of practice and are considered as the pretraining required by the board of pharmacy. Hopefully in the future the need for additional training post-graduation will lessen. Lastly, patient and physician awareness of pharmacist services needs to be improved, which can be accomplished by establishing relationships with primary care providers to increase patient referrals to the pharmacist. This may also pose an opportunity for professional pharmacy associations to advocate for strategies to improve relationships with other health professionals and to further educate patients. A study conducted by Snyder et al. revealed that physicians with face-to-face interactions with pharmacists allowed them to recognize pharmacists’ commitment to patient care, making them more likely to advocate for pharmacist-provided services [14]. Taking this extra step to meet with physicians enhances collaborative working relationships. Ultimately, this will lead to more opportunities for pharmacists to practice within their expanded scope in order to enhance patient care.

### 4.2. Limitations

This study had several limitations. First, the timing of distribution of this survey was amid the Coronavirus Disease 2019 (COVID-19) pandemic. Response rate and answer choices could have been affected by unprecedented circumstances that respondents were experiencing and may not be reflective of normal operations within the pharmacy. Also, while patient awareness of pharmacist-provided services was assessed, physician awareness was not. Additionally, the convenience sample was limited to members of professional pharmacy organizations and grocery store pharmacists due to the distribution methods. CPhA is the largest California pharmacist organization with community pharmacist membership so this was felt to be best representative of community pharmacists. Because the survey was optional, this led to a small sample size and may not represent all community pharmacists’ experiences within CA. As this was the research project of a PGY1 Community-Based Pharmacy Resident at Albertsons Companies, our survey was sent to all of their pharmacists in CA, leading to a large portion of respondents practicing in a grocery store-based setting. Similarly, the survey was distributed to the CA CPESN network, increasing the number of respondents from independent pharmacies. All findings were self-reported by the respondents, an inherent limitation in survey research. Due to the small sample size, we conducted limited subgroup analysis, which is another limitation of our study. Lastly, some survey items were partially completed since questions were optional. Authors deemed that partially completed surveys still held valuable information to contribute to the results.

## 5. Conclusions

This study identified factors that motivate or hinder California community pharmacists from providing patient care services and associated prescribing of medications. Pharmacists are highly interested and motivated to serve patients within their expanded scope of practice. Currently, ordering and administering vaccinations and prescribing naloxone are the primary focus of pharmacists. Few pharmacists bill Medicaid for their services. Opportunity exists for better engagement with more services and medication prescribing. Important barriers are lack of patient awareness of pharmacist-provided services, inability to incorporate services within a busy workflow, and lack of payment for services. Authors intend to re-distribute this survey within 1–2 years to re-evaluate how pharmacists’ motivation has changed and if barriers have been overcome.

## Figures and Tables

**Table 1:** Participant characteristics.

Characteristic	Percentage (No.)
**Additional training (*N* = 32)**	
PGY1 Residency	44% (14)
PGY2 Specialty Residency	13% (4)
Fellowship	9% (3)
Other	34% (11)
**Ethnicity (*N* = 139)**	
White	40% (56)
Hispanic or Latino	4% (6)
Black or African American	2% (3)
Native American	2% (3)
Asian or Pacific Islander	37% (52)
Other	5% (7)
Prefer not to specify	9% (12)
**Gender (*N* = 140)**	
Male	53% (74)
Female	41% (58)
Other	1% (1)
Prefer not to specify	5% (7)

**Table 2:** Patient groups served by pharmacies.

Characteristic	Percentage (No.)				
Patient groups served	0–25%	26–50%	51–75%	>75%	I don’t Know
Geriatrics (65 years and older)	15% (27/183)	33% (60/183)	39% (72/183)	11% (20/183)	2% (4/183)
Middle-age adults (40–64 years old)	18% (33/179)	59% (106/179)	18% (32/179)	1% (2/179)	3% (6/179)
Young adults (18–39 years old)	62% (110/177)	29% (51/177)	5% (9/177)	1% (2/177)	3% (5/177)
Pediatrics (under 18 years old)	84% (139/165)	12% (20/165)	1% (2/165)	0% (0/165)	2% (4/165)
Medicare & Medi-Cal (“Medi-Medi”) beneficiaries	34% (58/171)	28% (48/171)	19% (32/171)	12% (21/171)	7% (12/171)
Medi-Cal beneficiaries	32% (54/168)	25% (42/168)	23% (38/168)	15% (26/168)	5% (8/168)

**Table 3:** Pharmacy setting characteristics.

Prescriptions Dispensed per Week	Percentage (No.)
<500	14% (27)
500–1000	39% (73)
1001–1500	24% (44)
1501–2000	8% (14)
2001–2500	6% (11)
>2500	10% (18)
**Service Fee Billed**	**Percentage (SD, Range)**
Directly to Patient (*N* = 98)	16% (25.5, 0–100)
Commercial Payor (*N* = 96)	35% (27.9, 0–100)
Medicare (*N* = 98)	29% (23.4, 0–100)
Medi-Cal (*N* = 95)	22% (23.4, 0–100)
No bill or charge (*N* = 82)	15% (31.1, 0–100)

**Table 4:** Knowledge questions. These multiple-choice questions assess knowledge of providing patient care services. Correct answers are bolded.

Survey Question	Number of Individuals That Selected the Correct Answer
A pharmacist licensed in California who has completed the training required by the Board of Pharmacy can furnish the following medications (select all that apply): (a) Travel medications (b) Self-administered hormonal contraceptives (c) Routine vaccinations (d) Nicotine replacement therapies (e) Naloxone hydrochloride (f) HIV PrEP/PEP medications * (g) Chantix (varenicline) * (h) Buprenorphine (e.g., Suboxone) * (i) I don’t know	20% (34/168)
AB 1114 requires the rate of reimbursement for pharmacist services to be at ______ percentage of the fee schedule for physician services under the Medi-Cal program. (a) 50% (b) 65% (c) 70% (d) 85% (e) 100%	56% (25/45)
Reimbursement for pharmacist services provided to Medi-Cal beneficiaries is contingent upon (select all that apply): (a) Completion of a 2 h, AB 1114 continuing education session (b) Enrollment as an ordering, referring, and prescribing (ORP) provider with Medi-Cal (c) Payment of a yearly fee to the Department of Health Care Services (d) Completion of a PGY-1 residency (e) California licensure as an Advanced Practice Pharmacist	62% (51/82)

* These items were included as distractor options.
